# Primary sclerosing cholangitis with partial steroid responsiveness: a case report

**DOI:** 10.20407/fmj.2022-012

**Published:** 2022-07-22

**Authors:** Satoshi Yamamoto, Kazuo Inui, Yoshiaki Katano, Hironao Miyoshi, Kenji Notohara

**Affiliations:** 1 Department of Gastroenterology, Fujita Health University Bantane Hospital, Nagoya, Aichi, Japan; 2 Department of Gastroenterology, Yamashita Hospital, Ichinomiya, Aichi, Japan; 3 Department of Anatomic Pathology, Kurashiki Central Hospital, Kurashiki, Okayama, Japan

**Keywords:** Eosinophilia, IgG4-related sclerosing cholangitis, Primary sclerosing cholangitis, Eosinophilic cholangiopathy

## Abstract

A 69-year-old woman suspected to have IgG4-related sclerosing cholangitis causing bile duct stenosis was transferred from another hospital after diarrhea, eosinophilia, and eosinophilic infiltration were detected and prednisolone was prescribed. Additional biliary imaging suggested primary sclerosing cholangitis, but the IgG4 level and inferior bile duct stenosis were alleviated by steroid therapy, suggesting IgG4-related sclerosing cholangitis. Therefore, prednisolone was continued. Bile duct biopsy findings suggesting adenocarcinoma led to a diagnosis of pancreatoduodenectomy. The latter specimen only displayed evidence of primary sclerosing cholangitis, and prednisolone was discontinued. Intractable cholangitis necessitated left hepatectomy, after which serum alkaline phosphatase levels increased and eosinophilic colitis recurred. The reintroduction of prednisolone effectively managed the diarrhea but only temporarily reversed the alkaline phosphatase elevation. When histologic sections from resection specimens were compared, the hepatectomy specimen exhibited greater eosinophil infiltration than the earlier pancreatoduodenectomy specimen, suggesting eosinophilic cholangiopathy superimposed on primary sclerosing cholangitis.

## Introduction

Primary sclerosing cholangitis (PSC), characterized by the stricture of intrahepatic and extrahepatic bile ducts in biliary tract images, often gives rise to bile duct cancer or biliary cirrhosis.^1^ By contrast, IgG4-related sclerosing cholangitis (IgG4-SC) requires the additional presence of elevated serum IgG4 levels, and it responds well to steroid therapy. Distinguishing these diseases often is difficult, typically requiring cholangiography, endoscopic ultrasonography, and intraductal ultrasonography (IDUS) in addition to serum IgG4 measurements. We treated a patient with biliary tract imaging findings typical of PSC who experienced an atypical clinical course in which serum IgG4 levels were elevated, steroid therapy sometimes was beneficial, and eosinophilic enteritis also occurred. Additional histologic and immunohistologic examination of surgical specimens suggested eosinophilic cholangiopathy coexisting and possibly interacting with PSC.

## Case presentation

A 69-year-old woman presented at another hospital in August 2011 with epigastric pain. Her past history included cholelithiasis treated by cholecystectomy, and other prior surgeries included uterine myomectomy. Her family and social history were noncontributory. A common bile duct stone was detected ultrasonographically, and endoscopic retrograde cholangiography (ERC) in September disclosed stenosis of the distal common bile duct. Her elevated serum IgG4 level (201 mg/dL) raised suspicion of IgG4-SC, whereas epigastric pain and diarrhea occurred in association with an elevated peripheral eosinophil count of 8568/μL. A biopsy specimen from the duodenum revealed eosinophilic infiltration. Accordingly, the patient was diagnosed with eosinophilic enteritis at that hospital and treated with prednisolone (PSL; 30 mg/day). A biliary stent was placed, and the patient was transferred to our hospital at the end of September 2011.

The patient’s body temperature was 37.5°C, and her blood pressure was 157/85 mmHg. Her abdomen was flat and soft. No signs of anemia or jaundice were present. She reported mild epigastric pain but displayed no tenderness. Her serum IgG4 levels decreased from 201 mg/dL to 137 mg/dL. Her increased total white blood cell count was attributed to corticosteroid-induced demargination (Table 1).

**Figure 1:**
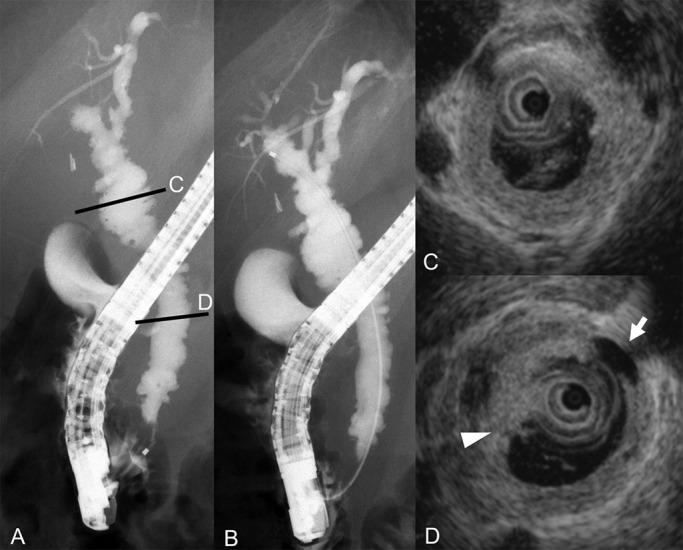
Findings of endoscopic retrograde cholangiography and intraductal ultrasonography on admission to our hospital. A–B) Endoscopic retrograde cholangiography revealed diverticulum-like outpouching of the distal common bile duct and a pruned-tree appearance of intrahepatic bile ducts. C–D) Intraductal ultrasonography demonstrated sludge (arrowhead) and diverticulum-like outpouching (arrow).

The ERC findings included diverticulum-like outpouching of the distal common bile duct and intrahepatic bile ducts with a pruned-tree appearance. Immunohistochemical examination of the distal common bile duct and duodenal papilla biopsy specimens revealed positivity for IgG4 in a few plasma cells. Because stenosis of the bile duct was apparently alleviated, the biliary stent was removed (Figure 1).

**Figure 2:**
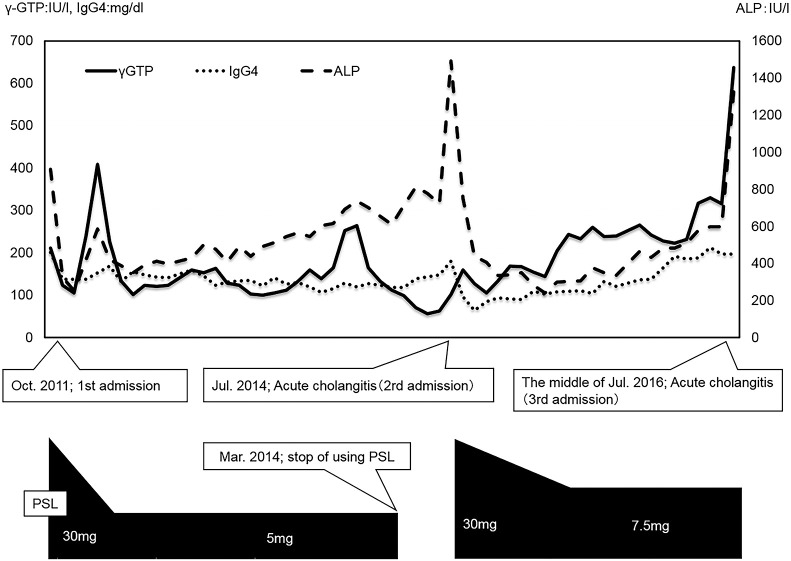
Clinical course. PSL, prednisolone

The patient’s PSL dose was decreased from 30 mg/day to 5 mg/day. The patient was considered to have PSC based on the characteristic biliary tract imaging findings. Because the elevated serum IgG4 levels and biliary duct stenosis of the biliary were alleviated by low-dose PSL administration, this dose was maintained until March 2014 (Figure 2).

The patient was readmitted in July 2014 with acute cholangitis (Figure 2), which was rapidly relieved by biliary stenting. PSL was reintroduced at 30 mg/day and then slowly reduced to 7.5 mg/day, and this dose was maintained for 2 years.

In July 2016, the patient was readmitted with acute cholangitis (Figure 2). Liver enzyme, direct bilirubin, and alkaline phosphatase (ALP) levels were elevated in serum, as was IgG4 levels (Table 2). Cholangitis improved with conservative treatment, but relapse occurred upon attempting oral nutrition. ERC was performed emergently, revealing purulent-appearing bile emerging from the papilla. A biliary stent was placed.

**Figure 3:**
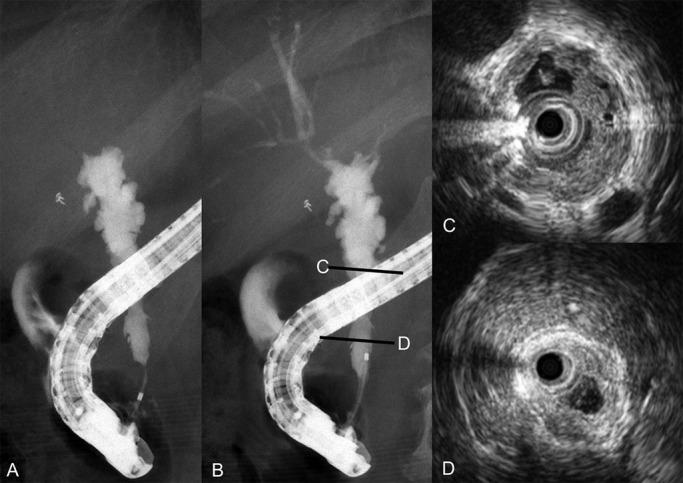
Endoscopic retrograde cholangiography and intraductal ultrasonography findings after clinical improvement. A–B) Endoscopic retrograde cholangiography revealed stenosis and outpouching. C–D) Intraductal ultrasonography depicted inhomogeneous wall thickness in the mid-portion of the common bile duct.

After clinical improvement, ERC and IDUS disclosed stenosis of the distal common bile duct and intrahepatic bile ducts. IDUS revealed variable wall thickness in the mid-portion of the common bile duct. A biopsy specimen from this area suggested adenocarcinoma, and a diagnosis of bile duct cancer associated with IgG4-SC was rendered (Figure 3). Because a bile duct biopsy specimen obtained above the cystic duct confluence did not reveal carcinoma, we proceeded with pancreatoduodenectomy in November 2016.

**Figure 4:**
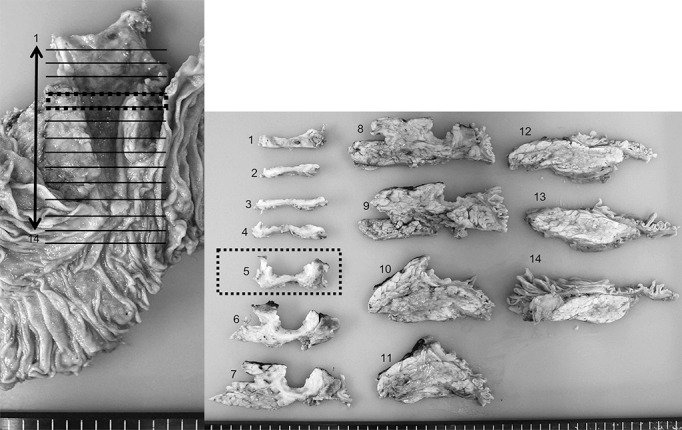
Macroscopic findings in the pancreatoduodenectomy specimen from November 2016. Extensive bile duct ulceration is evident (sites 1–10).

**Figure 5:**
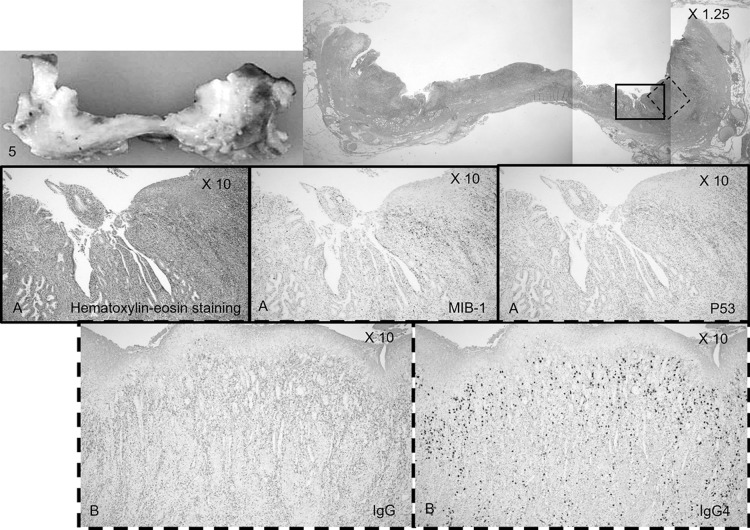
Microscopic findings in the pancreatoduodenectomy specimen (site 5). A) Hematoxylin–eosin staining and immunohistochemical staining revealing positivity for MIB-1 and negativity for p53 (original magnification, ×10). B) Immunohistochemical staining for IgG and IgG4. The IgG4-positive cell to IgG-positive ratio cell was small among plasma cells (original magnification, ×10).

Macroscopically, extensive common bile duct ulceration was evident in the resected specimen (Figure 4). Microscopically, cancer was absent, although regeneration-related biliary epithelial atypia with regeneration was evident. Immunohistochemical staining was performed to assess the biliary epithelium. MIB-1 staining was extensively positive, whereas p53 staining was positive only in some epithelial cells. PSC was diagnosed on the basis of fibrosis within granulation tissue, in which many IgG4-positive plasma cells were present but the ratio of IgG4-positive cells to IgG-positive plasma cells was small (Figure 5).

Approximately 1 year after surgery, worsening stricture of the left intrahepatic bile duct resulted in acute cholangitis requiring readmission (August 2017). Percutaneous transhepatic biliary drainage was attempted to treat the stricture, but the guidewire could not be advanced beyond the hilar portion of the duct. PSL was discontinued in September 2017, but left hepatectomy was required the following month because of intractable recurring cholangitis. Pathologic specimens from the left hepatectomy disclosed typical “onion-skin” fibrosis, suggesting PSC around the bile ducts.

In January 2018, serum ALP levels had risen from 1371 U/L to 3413 U/L without clinically evident cholangitis when diarrhea recurred. Eosinophilia (10,780/μL) was detected in peripheral blood, and colonoscopy disclosed mucosal erosion extending from the ileocecal junction to the ascending colon. A biopsy specimen from the erosion revealed eosinophil infiltration (>50 cells/high-power field [HPF]), indicating relapse of eosinophilic enteritis. Upon resuming PSL at 20 mg/day, diarrhea improved, and serum ALP levels decreased. Subsequently, serum ALP levels continued to fluctuate despite ongoing PSL therapy.

**Figure 6:**
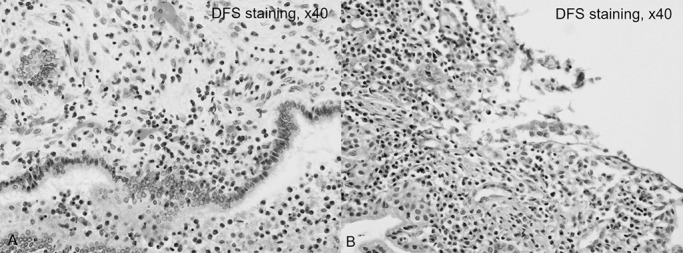
Eosinophilic infiltration in the left hepatectomy specimen versus the earlier pancreaticoduodenectomy specimen. A) Microscopic findings in the left hepatectomy specimen (without prednisolone administration). Many eosinophils (approximately 100 cells per high-power field) surround the bile duct (original magnification, ×40). B) Microscopic findings in the pancreatoduodenectomy specimen (with prednisolone administration). Eosinophil infiltration was less prevalent (50 cells per high-power field) than in the left hepatectomy specimen (original magnification, ×40).

Pathologic specimens from pancreatoduodenectomy (during PSL therapy; eosinophil infiltration, 50 cells/HPF) and subsequent left hepatectomy (without PSL; eosinophil infiltration, >50 cells/HPF) revealed an increase in eosinophil infiltration after discontinuing PSL, supporting the likelihood of eosinophilic cholangiopathy coexisting with PSC (Figure 6).

## Discussion

PSC was first described by Delbet in 1924,^2^ whereas the English term PSC was assigned much later by Schwartz in 1958.^3^ PSC is characterized by stricture of intra- and extrahepatic bile ducts, and it is often refractory to steroid therapy. Furthermore, PSC can lead to bile duct cancer or biliary cirrhosis. We diagnosed our present patient with PSC based on biliary imaging findings of diverticulum-like outpouching and a pruned-tree appearance of the intrahepatic biliary ducts. Steroid therapy, initiated at another hospital to treat eosinophilic gastroenteritis, both decreased serum IgG4 levels and alleviated stricture of the distal common bile duct, suggesting IgG4-SC.

Biopsy specimens taken from bile duct areas exhibiting inhomogeneous wall thickening were suggestive of adenocarcinoma, but because a bile duct biopsy specimen taken immediately above the confluence with the cystic duct did not display carcinoma, pancreatoduodenectomy could be performed. No bile duct cancer was identified in the resected specimen. IgG4-SC was suspected according to the clinical course, but pathologic findings including broad areas of duct ulceration were more consistent with PSC. Although many IgG4-positive plasma cells were detected immunohistochemically in granulation tissue undergoing fibrosis, the ratio of IgG4-positive cells to IgG-positive plasma cells was small, again supporting a diagnosis of PSC rather than IgG4-SC.

Tanaka reported that 216 of 435 patients with PSC displayed serum IgG4 elevation.^4^ Contrarily, bile duct ulcerations in PSC feature many IgG4-positive plasma cells, whereas peripheral portal areas contain few cells, unlike the findings in IgG4-SC.^5^

Steroid therapy displays no clear efficacy in PSC, and only ursodeoxycholic acid treatment has a proven benefit.^6^ Steroids may be helpful in an early stage of PSC, but prolonged steroid therapy increases risk of osteoporosis in patients with cholestasis.^7^ Biliary tract imaging in IgG4-SC rarely reveals features characteristic of PSC such as band-like strictures, beaded or pruned-tree appearances, or diverticulum-like outpouching.^8^ The biliary imaging findings in our patient were typical for PSC, specifically a pruned-tree appearance and diverticulum-like outpouching. In early assessments, she was considered likely to have IgG4-SC because IgG4 elevation improved and biliary tract imaging initially revealed decreased duct stenosis following PSL treatment. Subsequently, her biliary tract imaging findings worsened, and cholangitis recurred. We should have diagnosed PSC earlier based on the characteristics of the early images.

Two episodes of eosinophilic enteritis occurred during our patient’s course of PSC. The criteria for eosinophilia were defined by Fauci et al.^9^ In Japan, eosinophilia has been reported to occur in 27% of patients with PSC,^10^ whereas eosinophilia coexisting with PSC has rarely been reported in other countries. For example, Wiesner et al. detected eosinophilia in 1 of 50 patients with PSC.^11^ Eosinophilic disorders often affect the heart, lungs, and nervous system, but eosinophilia in nonparasitic bile duct disorders such as PSC is unusual in most localities.^12^ Mechanisms linking eosinophilia with PSC have not been clearly demonstrated, although Bartholomew proposed an allergic or hypersensitivity reaction as a cause.^13^ When we searched for PSC with eosinophilic colitis in PubMed covering the period of 1949 to 2021 using “PSC” and “eosinophil” as keywords, three patients apart from our current case were identified.^14–16^ The mean age of all four patients was 43±20 years, and the male:female ratio was 3:1. Among all patients, diarrhea, abdominal pain, abnormal liver function levels, and weight loss were observed in three, two, two, and one patient, respectively (overlap occurred). Three patients were treated with corticosteroids. One report, who was reported by Gunji, described the outcome of steroid therapy in detail. Specifically, eosinophilic enteritis and diarrhea improved, but PSC lesions and liver function abnormalities did not improve. Meanwhile, biliary tract imaging findings tended to worsen.^16^ Gunji suggested that eosinophilic granular proteins in affected tissues, such as eosinophil peroxidase, could stimulate eosinophil production in bone marrow and eosinophil migration to sites of inflammation.^16^

When eosinophilia accompanies diarrhea as observed in our patient, eosinophilic enterocolitis should be suspected, and colonoscopy should be performed. Such diarrhea usually responds to steroid therapy.

Eosinophilic cholangiopathy was first reported by Leegaard in 1980.^17^ On imaging, eosinophilic cholangiopathy features diffuse stricture of the bile ducts from the hilar bile ducts to the intrahepatic bile ducts, and stricture of the distal bile duct can also be present.^18,19^ Accordingly, pathologic findings and the clinical course are important for diagnosing eosinophilic cholangiopathy given the difficulty of distinguishing PSC from eosinophilic cholangiopathy using imaging findings alone. Sclerosing cholangitis secondary to eosinophilic cholangiopathy has also been reported,^18,20^ but to our knowledge, eosinophilic cholangiopathy coexisting with PSC has not been described. We suspected eosinophilic cholangiopathy coexisting with PSC in our patient for two reasons. First, eosinophilic enteritis accompanied by peripheral blood eosinophilia and ALP elevation occurred approximately 3 months after left hepatectomy, and corticosteroids were not being administered at this time. Second, microscopic findings from hepatectomy, performed in the absence of PSL, included more eosinophils than present in the earlier pancreatoduodenectomy specimen obtained during PSL treatment.

Sclerosing cholangitis secondary to eosinophilic cholangiopathy is particularly difficult to differentiate from PSC in Japan, where many patients have eosinophilia coexisting with PSC.^10,20^ Watanabe reported hepatic eosinophil infiltration coexisting with PSC.^21^ Activated eosinophils are reported to produce a cationic eosinophil granule protein termed major basic protein, as well as various cytokines including transforming growth factor beta.^22,23^ Horiuchi reported that such cytokines can induce inflammation and fibrotic changes in bile ducts.^20^

To our knowledge, eosinophilic cholangiopathy coexisting with PSC has not been reported previously. The interactions between these entities are unclear, but immune system abnormalities may be pathogenetically important.

## Figures and Tables

**Table1:** Laboratory findings on the first admission

**Peripheral blood cell examination**		
WBCs	13,800	/μL
Eosinophils	138	/μL
RBCs	430×10^4^	/μL
Hb	12.4	g/dL
Ht	38.1	%
Plt	35.8×10^4^	/μL
ALP	327	IU/L
γGTP	123	IU/L
Amy	117	U/L
Na	141	mEq/L
K	4.1	mEq/L
Cl	103	mEq/L
BUN	16	mg/dL
**Biochemical determinations**		
T-bil	0.6	mg/dL
D-bil	0.1	mg/dL
AST	19	IU/L
ALT	20	IU/L
LDH	266	IU/L
Cr	0.8	mg/dL
CRP	0.65	mg/dL
IgG4	137	mg/dL
CEA	2.7	ng/dL
CA19-9	0.1	U/mL

WBCs, white blood cells; RBCs, red blood cells; Hb, hemoglobin; Ht, hematocrit; Plt, platelets; T-bil, total bilirubin; D-bil, direct bilirubin; AST, aspartate aminotransferase; ALT, alanine aminotransferase; LDH, lactate dehydrogenase; ALP, alkaline phosphatase; γGTP, γ-glutamyl transpeptidase; Amy, amylase; Na, sodium; K, potassium; Cl, chloride; BUN, blood urea nitrogen; Cr, creatinine; CRP, C-reactive protein; IgG4, immunoglobulin G4; CEA, carcinoembryonic antigen; CA19-9, carbohydrate antigen 19-9

**Table2:** Laboratory findings on the third admission

**Peripheral blood cell examination**		
WBCs	9 500	/μL
Eosinophils	190	/μL
RBCs	472×104	/μL
Hb	11.9	g/dL
Ht	39.1	%
Plt	39.4×104	/μL
ALP	1 329	U/L
γGTP	637	IU/L
Amy	71	U/L
Na	140	mEq/L
K	4.5	mEq/L
Cl	103	mEq/L
BUN	11	mg/dL
**Biochemical determinations**		
T-bil	1.2	mg/dL
D-bil	0.8	mg/dL
AST	149	IU/L
ALT	165	IU/L
LDH	333	IU/L
Cr	1.03	mg/dL
CRP	2.53	mg/dL
IgG4	197	mg/dL
CEA	4.6	ng/dL
CA19-9	0.1	U/mL

WBCs, white blood cells; RBCs, red blood cells; Hb, hemoglobin; Ht, hematocrit; Plt, platelets; T-bil, total bilirubin; D-bil, direct bilirubin; AST, aspartate aminotransferase; ALT, alanine aminotransferase; LDH, lactate dehydrogenase; ALP, alkaline phosphatase; γGTP, γ-glutamyl transpeptidase; Amy, amylase; Na, sodium; K, potassium; Cl, chloride; BUN, blood urea nitrogen; Cr, creatinine; CRP, C-reactive protein; IgG4, immunoglobulin G4; CEA, carcinoembryonic antigen; CA19-9, carbohydrate antigen 19-9
